# Clinicopathological characteristics and prognosis of gastrointestinal stromal tumors containing air-fluid levels

**DOI:** 10.1371/journal.pone.0261566

**Published:** 2021-12-17

**Authors:** Tianzhu Liu, Gao Lin, Hui Peng, Lesheng Huang, Xiaosong Jiang, Hongyi Li, Kaili Cai, Jinghua Jiang, Lei Guo, Xiaohua Du, Jiahui Tang, Wanchun Zhang, Jun Chen, Yongsong Ye

**Affiliations:** 1 Department of Radiology, Guangdong Hospital of Traditional Chinese Medicine, Zhuhai, China; 2 Department of General Surgery, Guangdong Hospital of Traditional Chinese Medicine, Zhuhai, Guangdong, China; 3 Department of Pathology, Guangdong Hospital of Traditional Chinese Medicine, Zhuhai, Guangdong, China; 4 Department of Radiology, Guangdong Hospital of Traditional Chinese Medicine, Guangzhou, Guangdong, China; UKSH Campus Lübeck, GERMANY

## Abstract

An air-fluid level within a gastrointestinal stromal tumor (GIST) is unusual and indicates the presence of a fistula within the lumen of the GI tract. Until recently, the optimal management of such patients was not clear-cut. This retrospective study investigated the clinicopathological characteristics, surgical procedures, pre-and post-operative management, and prognosis of patients with GIST containing an air-fluid level. Data of GIST patients, spanning 5 years, including 17 GIST patients with air-fluid levels in the experimental group and 34 GIST patients without air-fluid levels in the control group, were retrieved from two hospitals in China. The clinicopathological characteristics, types of surgery, management, and clinical outcomes of GIST patients were compared between the two groups. GISTs containing air-fluid levels were significantly different from GISTs without air-fluid levels regarding tumor morphology, NIH risk category, invasion of adjacent organs, and necrosis or ulceration. Most GIST patients with air-fluid levels (14/17, 82.4%) received open surgery, significantly higher than the 20.6% in the control group. Targeted therapy with Imatinib mesylate (IM) was implemented in all GIST patients in the experimental group (17/17, 100%); markedly higher than those (3/34, 8.8%) in the control group. During follow-up, recurrence and death rates (5.9% and 5.9%) in the experimental group were higher than those (2.9% and 0%) in the control group. Open surgery is commonly performed in GIST patients with air-fluid levels who also require targeted therapy with IM. The Torricelli-Bernoulli sign could be a risk factor, adversely affecting the patient’s prognosis.

## Introduction

A gastrointestinal stromal tumor (GIST), the most common mesenchymal tumor of the gastrointestinal (GI) tract, is derived from cells presenting morphological and immunophenotypic similarities with Cajal cells [1]. GIST can occur throughout the entire GI tract, with the stomach and the small intestine as the two most common sites, accounting for 60% and 30%, respectively [2]. The annual incidence of GIST is estimated to be between 11 and 14.5 cases per million, with GIST accounting for between 1% and 3% of all GI tumors [3]. Most patients (69%) with primary GIST arising in the GI tract present with clinical symptoms. In contrast, a small proportion of GISTs are asymptomatic, and the tumor without symptoms is usually incidentally detected during imaging, by palpation, at surgery for other conditions, or at autopsy [4]. The main therapeutic approaches for patients with GISTs are surgery and targeted drug therapy, while other less common treatments include chemotherapy, ablation and embolization, and radiation therapy [2].

Imaging examinations, including computed tomography (CT), magnetic resonance imaging (MRI), endoscopy, abdominal ultrasound, and positron emission tomography (PET), can be used to identify GISTs, of which CT scan is considered the best imaging modality in identifying the tumor location, invasion or metastasis to near structures. Notably, air-fluid levels or bubbles on CT or MRI scans were detected in a small proportion of patients with GISTs [5, 6]. Although detecting air-fluid levels within GIST is uncommon, such imaging features may indicate a severe condition in GIST patients. Case reports have documented the occurrence of air-fluid levels or bubbles, but no comparative studies of the clinicopathological characteristics, imaging, and histopathological features, treatments, and clinical outcomes have been conducted. To date, the optimal management of such patients has not been established.

In this study, we retrospectively enrolled GIST patients from two hospitals in Guangdong, China spanning five years to investigate the clinicopathological characteristics, surgical procedures, pre-and post-operative management, and prognosis of patients with GIST containing air-fluid levels or bubbles. The results may improve our GIST knowledge and assist oncologists/gastroenterologists in better managing patients with air-fluid levels.

## Materials and methods

### Patients and study design

The Ethics and Research Committee of Guangdong Hospital of Traditional Chinese Medicine Zhuhai, 519000, China approved the study (No. ZE2021-021-01); all procedures were performed per the relevant guidelines and regulations. Informed consent for the use of medical records was obtained from all living participants.

In this study, we retrospectively reviewed the medical records of patients with primary GIST admitted to the Guangdong Hospital of Traditional Chinese Medicine (Zhuhai, 519000, China) and Zhuhai Hospital affiliated to Guangdong Hospital of Traditional Chinese Medicine (Zhuhai, Guangdong, China) over five years between January 2015 and December 2020. Inclusion criteria were: (1) Primary GIST confirmed by post-operative histological examinations of surgically resected tissues or biopsy specimens; (2) Had pre-operative CT or MRI scans performed. Exclusion criteria were: (1) the presence of comorbid disorders or metastases, making the patient ineligible for surgery, (2) coexisting malignancies; (3) incomplete pathology reports.

The enrollment process is schematically shown in S1 Fig. A total of 442 patients were evaluated for their eligibility, 367 patients were excluded, of which 261 patients underwent ultrasound examination without CT or MRI scans for evaluation of lesions smaller than 1 cm in diameter. Consequently, 75 patients were enrolled in this study. Depending on whether or not air-fluid levels or air bubbles were observed on MRI or CT images, the patients were divided into two groups: those with air-fluid levels or air bubbles as the experimental group (n = 17) and those without air-fluid levels or air bubbles as the control group (n = 58). To improve comparability between the two groups, propensity score matching (PSM) was performed with the statistical software SPSS version 26.0 (IBM Corp, Armonk, NY, USA). Propensity scores were obtained using a logistic regression model with age, gender, with or without symptoms to provide a one-to-two match between the experimental and control groups, and the caliper was 0.02. Accordingly, 17 patients in the experimental group and 34 in the control group met the matching condition (S2 Fig).

### Imaging examinations

Pre-operative imaging procedures were employed to detect GIST in all the enrolled patients, including computed tomography (CT) scan in 16 patients, magnetic resonance imaging (MRI) in one patient in the experimental group, and CT scan in 34 patients in the control group. CT scans were performed on a 320-slice spiral CT machine (Aquilion ONE, Canon Japan, Tokyo) and a dual-source CT machine (Definition, Siemens German, Berlin). Before the CT examination, patients fasted for 12 hours and drank 1000–1200 ml of water. Scanning was performed in the standard supine position. The scanning conditions were as follows: the width of the collimator was 0.5 m, with a pitch of 1.0 mm; the thickness of the reconstructed slice was 5 mm, and the gap was 5 mm. In enhanced scanning mode, 100 ml iopromide (300 mg/ml) (Bayer Schering Pharma AG Co. Ltd., Leverkusen, Germany) was injected at a dose of 1–2 ml/kg and flow rate of 3.0 ml/s into the median cubital vein. Arterial phase scanning was performed at 20–25 seconds after injection, and venous phase scanning was performed at 55–60 seconds. On completing the scanning procedures, the reconstruction determined the correlations between the lesions and ambient tissues using maximum intensity projection (MIP) and multi-planar reconstruction (MRP).

The CT and MRI images were reviewed and assessed by two experienced radiologists (L.H & T.L) who had been practicing radiology for 14 and 10 years, respectively. The assessment included the location, size, shape, necrotic area, margins, and growth pattern of the tumor.

### Histopathology and immunohistochemistry

Histopathology was performed using resected or biopsy specimens. The size of each lesion was measured in the biopsy specimens. If more than one lesion was identified in an individual patient, the lesion containing an air-fluid level in the experimental group and the largest lesion in the control group were measured. The histological characteristics included tumor necrosis, surface erosion or ulceration, and cell type (spindle cell type, epithelioid cell type, and mixed spindle-epithelioid type). The mitotic index was defined as the number of mitotic figures per 50 high-power fields (HPFs) under light microscopy.

Immunohistochemistry (IHC) was performed to examine the expression levels of interest proteins. Seven primary antibodies were purchased from Roche Biological Technology (Roche, Shanghai, China), including mouse anti-CD117 monoclonal antibody (1:100 dilution), a rabbit anti-CD34 polyclonal antibody (1:200 dilution), a mouse anti-DOG1 monoclonal antibody (1:200 dilution), a mouse anti-SMA monoclonal antibody (1:150 dilution), a mouse anti-S100 monoclonal antibody (1:50 dilution), a mouse anti-desmin monoclonal antibody (1:100 dilution), and a rabbit anti-Ki-67 monoclonal antibody (1:100 dilution).

Based on tumor size and mitotic index, tumor risk classes were estimated per the modified National Institutes of Health (NIH) classification system proposed by Joensuu [7], in which tumor risk classes were classified into very low-risk, low-risk, intermediate-risk, and high-risk groups.

### Patient follow-up

Follow-up data were obtained through phone calls with the patients or their close family members and outpatient clinic visits after discharge. Intermediate- and high-risk patients were scheduled to undergo abdominal CT scan or MRI every three months for the first three years after the operation, then every six months until the fifth year. Very low- and low-risk patients underwent CT or MRI examinations every 6–12 months for five years after the operation. The last follow-up assessment of patients in the study was on December 31, 2020.

### Statistical analysis

All statistical analyses were performed using SPSS version 26.0 (IBM Corp, Armonk, NY, USA). The distribution diagram was plotted using GraphPad Prism version 8.0.1 (GraphPad Software., San Diego, CA, USA). Data were presented as the median, mean, percentage, and number of cases. *Pearson* correlation analysis was used to evaluate the correlation between tumor size in maximal diameter (MD) and necrosis area, with the normal distribution test performed before the analysis. The baseline characteristics and their differences between groups were compared using *Pearson’s* chi-square test for proportions. Student t-test was applied for continuous variables, Chi-square test for unordered categorical variables, and Wilcoxon rank-sum test for ordered classification variables.

## Results

### Demographic and clinical characteristics of GIST patients in the experimental group and control group

GIST patients’ demographic and clinical characteristics, including 17 patients with air-fluid levels or air bubbles on CT/MRI images in the experimental group and 34 patients in the control group, are summarized in Table 1. The incidence of GIST with air-fluid levels or air bubbles in GIST patients was 3.85% (17/442). The experimental group’s median age was 59 years (range, 39–81 years) and 60 years (range, 31–93 years) in the control group. Gender distribution analysis showed that 58.8% of patients in both groups were males, and the age and gender were matched between the two groups. Eleven symptomatic patients accounted for 64.7% of patients in the experimental group, similar to 69.6% in the control group. Abdominal pain was identified as the most common symptom in the experimental group (6/17, 35.3%) and the control group (20/34, 58.8%), followed by gastrointestinal bleeding presenting as melena in the experimental group (3/17, 17.6%) and control group (4/34, 11.8%).

**Table 1:** Demographic and clinical characteristics of GIST patients in the experimental group and control group.

Characteristics	GIST patients	
	*Experimental group*	*Control group*
Gender		
Male	10 (58.8%)	20 (58.8%)
Female	7 (41.2%)	14(41.2%)
Age (years)		
<40	2	4
40 to <50	2	9
50 to <60	5	4
60 to <70	3	13
70 to <80	3	1
≥80	2	3
Age range (years)	39–81	31–93
Median age (years)	59	60
Symptoms		
Abdominal pain	6	20
Gastrointestinal bleeding	3	4
Abdominal mass	1	0
Abdominal distension	1	0
Anemia	1	0
Accidental finding	5	10

### Radiological features of GIST patients in the experimental group and control group

**Fig 1:**
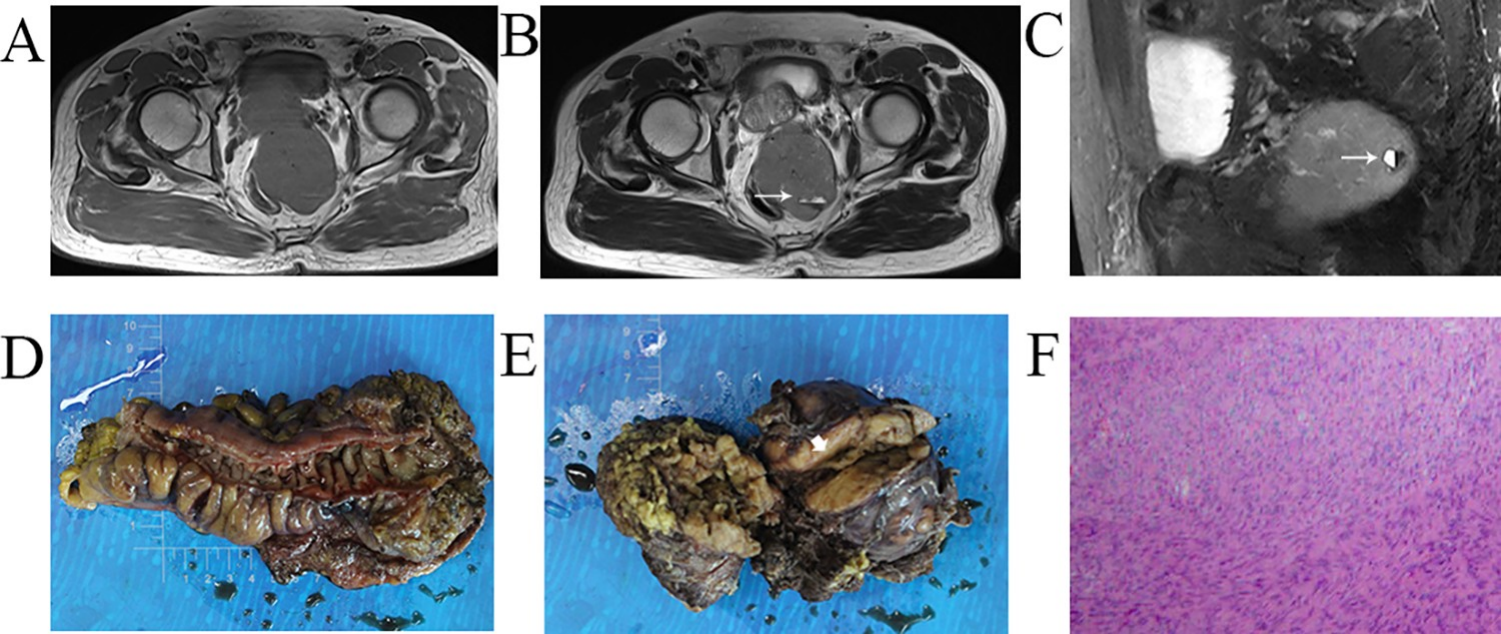
Representative images of computed tomography scans and histological examinations of patients with an air-fluid level. T1WI, T2WI, and Fat-suppression sequence showed (A) the mass and (B, C) air-fluid level (white arrow);.The tumor was extramural to (D) the colon wall and (E) the cavity (white arrowhead), which can be observed in the specimen; (F) Histopathological result of H&E staining showing the spindle cells (magnification, x100).

CT scan was performed in 34 (100%) patients in the control group and 16 (94.1%) in the experimental group. One patient (5.9%) in the experimental group underwent an MRI scan (Fig 1). The imaging features of GIST patients in the two groups are summarized in Table 2. CT and MRI imaging provided an explicit definitive diagnosis of GIST in 12 patients in the experimental group, an antidiastole of GIST in three patients, a misdiagnosis of neuroendocrine tumor in one patient, and a misdiagnosis of gastric carcinoma in one patient. Of 17 GIST cases in the experimental group, seven tumors did not have a confirmed site of origin and had ill-defined margins. CT imaging provided a definitive diagnosis of GIST in 19 patients in the control group, an antidiastole of GIST in 8 patients, misdiagnosis of neuroendocrine tumor in four patients, hemangioma in one patient, solitary fibrous tumor in one patient, and benign tumor in one patient. Among 34 GIST cases in the control group, ten tumors did not have a confirmed site of origin. Wilcoxon rank-sum test was performed, and there were no significant differences in the categorical variables between the two groups (*P* > 0.05).

**Table 2:** Radiological and histopathological characteristics of GIST patients in the experimental group and control group.

Variables	GIST patients		
	*Experimental group*	*Control group*	*P-value*
Tumor location			
Stomach vs Duodenum vs jejunum vs ileum vs mesentery vs rectum vs undefined	8 vs 3 vs 0 vs 4 vs 1 vs 1 vs 0	21 vs 4 vs 1 vs 4 vs 0 vs 1 vs 3	0.427
Tumor size (maximal diameter, cm)			
Range	2.6–16	0.8–22	0.254
Mean ± SD	8.71±4.17	6.50±5.59	
Tumor size (cm)			
≤5 vs 5–10 vs ≥10	4 vs 7 vs 6	21 vs 4 vs 9	
Necrotic area (maximal diameter, cm)			
Range	0.6–10.3	NA	
Mean ± SD	2.12±2.64		
Growth pattern			
Exophytic vs. Intraluminal Combined vs. undefined	2 vs 9 vs 6 vs 0	5 vs 16 vs 11 vs 2	0.758
Tumor margins			
Well-defined vs. ill-defined	8 vs 9	20 vs 14	0.426
Tumor shape			
Smooth/mildly lobulated vs. irregular	8 vs 9	26 vs 8	0.036
Cell type			
Spindle vs. epithelioid vs. mixed	13 vs 0 vs 4	29 vs 2 vs 3	0.235
Mitotic count (in 50 hPFs)			
≤5 vs >5	11 vs 6	27 vs 7	0.256
NIH risk categories			
Very low vs. low vs. intermediate vs. high	0 vs 3 vs 4 vs 10	6 vs 12 vs 3 vs 13	0.033
Local adhesion or invasion to adjacent organs			
Absence vs. presence	8 vs 9	32 vs 2	<0.0001
Necrosis or ulcer	17(100%)	10(29.4%)	<0.0001
Metastasis			
Absence vs presence	15 vs 2	33 vs 1	0.207

**Note**: GIST, gastrointestinal stromal tumor; HPFs, high power fields; NIH, National Institutes of Health. NA, not applicable.

CT/MRI imaging detected necrotic areas in the experimental group but not in the control group. The median tumor size was 8.7 cm (range, 2.6–16 cm), and the necrotic area was 2.1cm (range, 0.6–10.3 cm). The tumor size and the necrotic area size were normally distributed, and the Pearson correlation analysis indicated a significant correlation between tumor size and necrotic area (*P* = 0.003, correlation coefficient = 0.682) (S3 Fig).

### Histopathological and immunohistochemical findings in the experimental group and control group

The histopathologic features of GIST patients in the experimental and control groups are summarized in Table 2. There was no significant difference between two groups in the tumor location, size, growth pattern, tumor margins, cell type, and mitotic count of GISTs. Notably, a comparison of histopathological findings between the two groups revealed statistical differences in tumor shape (*P* = 0.036), NIH risk categories (P = 0.033), invasion to adjacent organs (*P* <0.0001), and necrosis or ulcer in the microscope (*P* <0.0001) (Table 2).

The immunohistochemical findings in the experimental and control groups are summarized in Table 3. Statistical analysis showed no significant differences in these immunohistochemical characteristics between the two groups.

**Table 3:** Immunohistochemical characteristics of GIST patients in the experimental group and control group.

Variables	GIST patients		*P-value*
	*Experimental group*	*Control group*	
	(n = 16)	(n = 34)	
CD117	100%(16/16)	100%(34/34)	No value
CD34	87.5%(14/16)	88.2%(30/34)	0.941
DOG1	87.5%(14/16)	100.0%(34/34)	0.098
SMA	31.3%(5/16)	38.2%(21/34) and 5 cases lost	0.795
S100	12.5%(2/16)	5.9%(1/34) and 1 case lost	0.508
desmin	18.8%(3/16)	23.5%(8/34) and 1 case lost	0.666
Ki-67≥10%	31.3%(5/16)	11.8%(4/34)	0.085

### Surgical procedures, post-operative treatments, and clinical outcomes in the experimental group and control group

All GIST patients in the two groups underwent surgical treatment, including traditional open surgery or minimally invasive surgical procedures. Open surgery was performed in 82.4% in the experimental group, considerably higher than the 20.6% in the control group. Laparoscopic surgery was performed in 17.6% of patients in the experimental group, lower than 55.9% in the control group. No patient in the experimental group and eight patients (23.5%) in the control group underwent endoscopic submucosal dissection (ESD). Among patients who underwent tumor resection, R0 and R1 resection rates were 92.3% and 5.9%, in the experimental group, 67.6%, and 8.8%, respectively, in the control group. No significant complications occurred in the two groups. The postoperative hospital stay ranged between 4 and 14 days (mean, 8.8 days) in the experimental group and 1 and 17 days (mean, 7.6 days) in the control group.

The median follow-up period was 19 months (range, 3–42 months) in the experimental group and 13.8 months (range, 3–38 months) in the control group. Of two patients with liver metastases in the experimental group, one patient was treated with neoadjuvant chemotherapy for six months, underwent open surgery, followed by outpatient treatment with imatinib mesylate (IM) after discharge. Tumor metastases were found at the 3-and-half-year follow-up. The other patient underwent open surgery followed by treatment with IM and follow-up after discharge. No metastases were found at the 2-year follow-up. One patient with a tumor located in the rectum refused postoperative treatment with IM and had a recurrence after two and a half years. A cystostomy was performed as palliative care, and the patient died from acute gastrointestinal bleeding six months later. The other 13 patients were treated with IM post-operatively and remained alive without recurrence and metastasis until their last clinical follow-up in December 2020. Only three patients required long-term IM treatment based on the relatively high proportion of very low or low-risk categories in the control group. All 34 patients underwent complete tumor resection, and one patient suffered a recurrence; 33 patients had no recurrence or metastasis.

## Discussion

This study of clinicopathological characteristics, treatments, and prognosis of patients with GIST containing air-fluid levels or bubbles has the following significant novel findings: (1) The necrotic area was positively correlated with tumor size in GIST patients with air-fluid levels or bubbles; (2) GISTs containing air-fluid levels showed significant differences in tumor morphology, NIH risk category, invasion to adjacent organs, and necrosis or ulcer compared with GISTs without air-fluid levels or bubbles; (3) Open surgery was more commonly performed in GIST patients with air-fluid levels, accounting for 82.4%, in contrast to 20.6% in GIST patients without detection of these features; (4) A targeted therapy with IM was needed in all GIST patients with air-fluid levels; (5) During follow-up, recurrence rate was 5.9% in GIST patients with air-fluid levels, higher than 2.9% GIST patients without this feature; (6) T-B sign could be a characteristic feature and risk factor associated with poor prognosis of GIST patients with air-fluid levels.

Regarding the high ratios of irregular shape, NIH risk category, invasion of adjacent organs, and necrosis or ulcers in GISTs containing air-fluid levels, we were inclined to think that GISTs containing air-fluid levels may exhibit more aggressive biological appearances [8] and supplementary rapid growth than those without air-fluid levels. During tumor growth, a tumor mass with supplementary rapid growth quickly outstrips its vasculature and lacks oxygen and nutrients, which could cause the necrotic areas of GIST and could explain the positive correlation of a necrotic area with tumor size. The malignant characteristics of GISTs with air-fluid levels (e.g., irregular shape, invasion of adjacent organs, necrosis) are apparent in pre-operative CT or MR scans. These characteristics may assist surgeons in making treatment decisions, including taking more aggressive and extensive surgery programs and targeted therapy with IM. Notably, although most of the patients in the experimental group underwent open surgery and targeted therapy, the recurrence rate was still higher than that in the control group (5.9% vs. 2.9%), which could be explained by the relationship between tumor burden, tumor size, curability, drug resistance and prognosis [9]. In this study, the tumor size between the experimental and control groups differed (8.71 ± 4.17 vs. 6.50 ± 5.59). Tumor size is the most commonly used variable affecting curability, drug resistance, and prognosis, with larger tumor size associated with higher tumor burden [9]. According to Goldie Coldman’s hypothesis of drug resistance [10], the probability that cancer contains drug-resistant clones depends on the mutation rate and the tumor size. With a given mutation rate, tumor size is considered the critical determinant in predicting the presence of drug-resistant mutations, and the drug resistance may decrease or even abrogate the therapeutic effect of targeted therapy [9]. Although it remains unclear whether GIST with air-fluid level is positively correlated with tumor burden, it has been noted that GIST with air-fluid level occurred in larger GIST. This finding may reflect the malignant and invasive biological characteristics of GIST with air-fluid levels.

**Fig 2:**
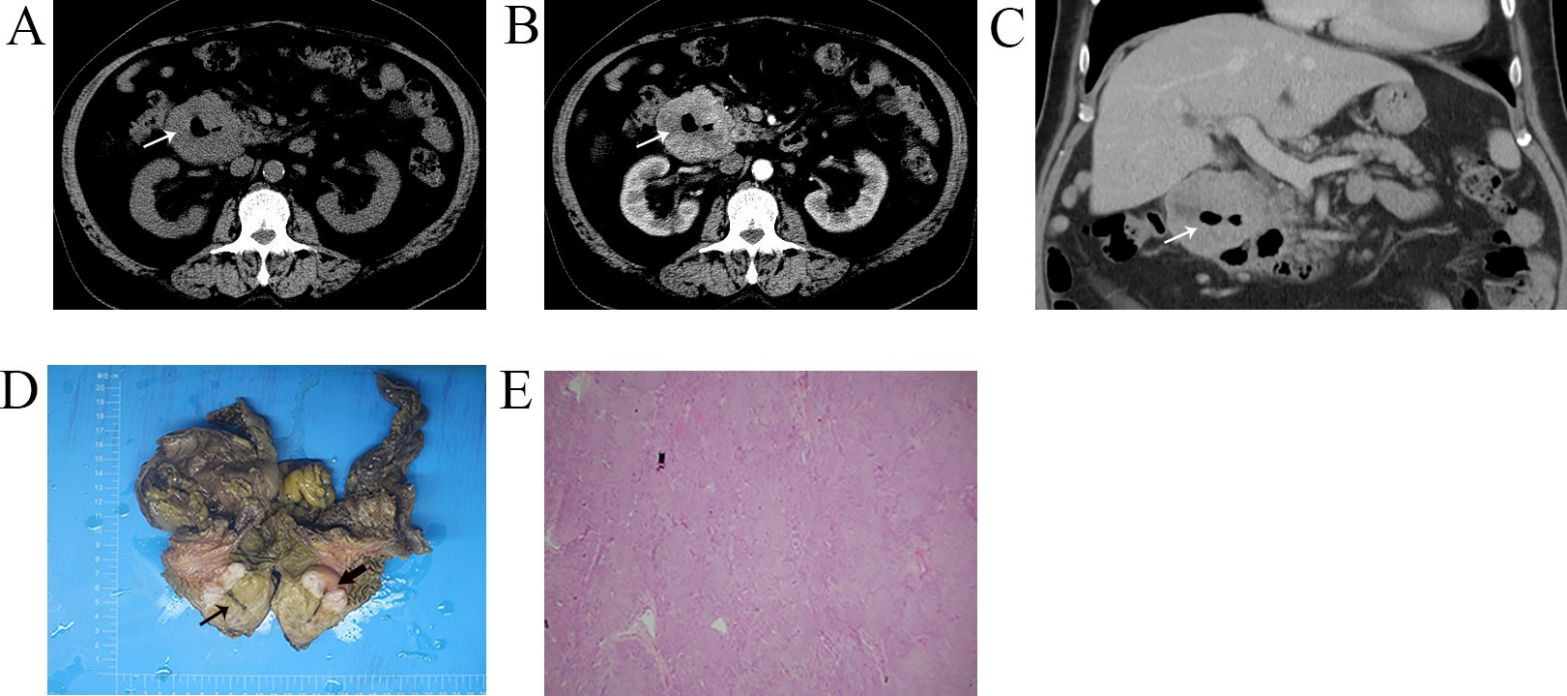
Representative CT images and histological examinations of duodenal GIST with an air-fluid level. (A-C) Enhancement of duodenal GIST and air-fluid level within the mass (white arrow); (D) The fistula (black arrow) and fistula opening (black arrowhead) between the wall of duodenum and mass;. (E) Histopathological result by H-E staining showed the spindle cells (magnification, x100).

**Fig 3:**
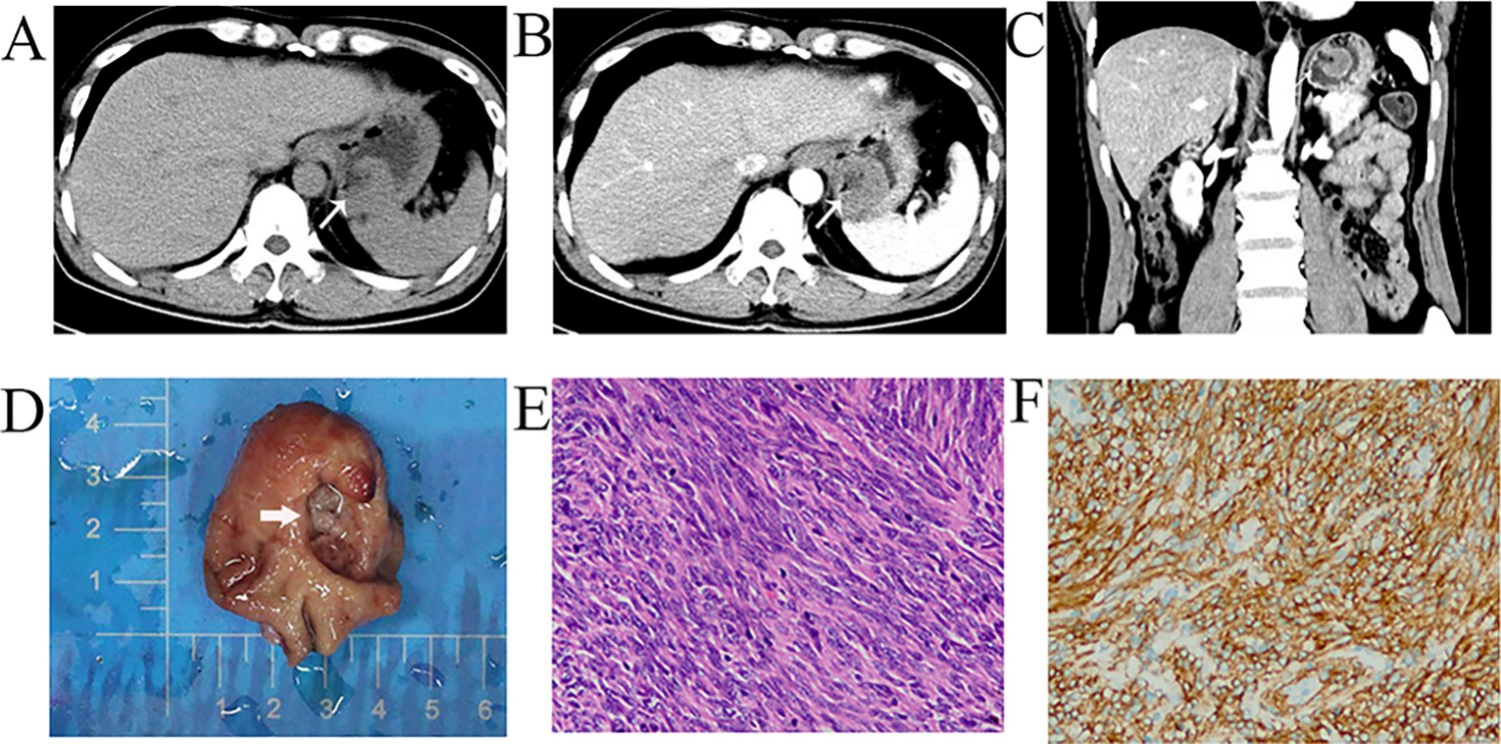
Representative CT images and histological examinations of GIST with an air-fluid level in the stomach. (A, B) Enhancement of tumor located in the fundus of the stomach) and air within the mass (white arrow, A & B); (C) The fistula between gastric cavity and tumor in CT reconstruction coronal position (white arrow) and (D) specimen (white arrowhead); (E) Histopathological result by H&E staining showed the spindle cells (magnification, x200); (F) Immunohistochemical staining (magnification, x200) showed positive for CD117.

It is worth noting that in our study, the pressure of a necrotic cavity may be equal to that of the intestinal tract in some GIST cases, making the liquid unable to flow out or flow into the necrotic cavity of GIST (S4 Fig). This condition was first described and interpreted by Fortman [6] and termed as the “Torricelli-Bernoulli sign (T-B sign).” Since 1999, the T-B sign has been reported in patients with GISTs [5, 11–13]. Regarding the occurrence of air-fluid levels or bubbles in GISTs (Figs 2, 3 and S5–S7), there is a divergence of findings in previous studies. Some clinicians consider the T-B sign a manifestation [13–18], while others consider it a common risk factor adversely affecting prognosis in association with recurrence of GIST [7, 19–26]. Moreover, rupture, perforation, or fistula formation in GISTs have not been well defined, and their definitions remain vague and controversial [27–29]. Based on our findings and those of others in the literature, we are inclined to think that the T-B sign was the process of fistula formation due to necrosis and ulceration of GIST and destruction of the intestinal mucosa.

Most previous studies indicated that GIST rupture was a risk factor for recurrence and poor prognosis [7, 19–26]. However, these studies did not distinguish whether the tumor ruptured into the lumen and formed a tumor-intestine wall fistula or ruptured out of the lumen and involved the peritoneum and abdominal cavity. Based on the imaging and histopathological findings of this study, we found that the ratios of the irregular shape of the tumor, invasion of adjacent organs, and NIH higher risk categories were significantly higher in GISTs with air-fluid levels compared with GISTs without air-fluid levels. The findings suggest that GISTs with the T-B sign have a faster growth rate, stronger invasive capabilities, and higher-risk categories.

This study has several limitations. Primarily, the sample size of patients with GIST with air-fluid levels or bubbles is relatively small because of the extremely low incidence of this condition. Furthermore, this is a retrospective study, and patient selection bias may have occurred. Also, the follow-up period was not long enough in a proportion of patients. Finally, we have not identified the decisive factor in the formation of GIST with air-fluid levels or bubbles.

## Conclusion

This retrospective study has shown that the T-B sign is relatively rare but could be a characteristic sign of GIST. Furthermore, the T-B sign may indicate the process of fistula formation due to the necrosis and ulceration of GIST and destruction of the intestinal mucosa, and it may also be a potential adverse prognostic factor for GIST. Thus, targeted therapy with IM is needed, and extensive surgery such as extended lymph node dissection or resection may be necessary. Because of the relatively high rate of metastasis, regular and long-term follow-up care is a sine qua non.

## Supporting information

S1 Fig Schematic diagram of patient enrollment. (PDF)

S2 Fig The plots of propensity scores before and after matching. (PDF)

S3 Fig Linear regression showing the relationship between tumor size and necrotic area in GIST patients with air-fluid levels. The analysis showed a significant correlation between tumor size in maximum diameter and necrotic area in GIST patients with air-fluid levels.
(TIF)

S4 Fig Proposed fistula formation in GIST patients prone to necrosis and ulceration. (A, B) Fistula formation between the tumor and the intestinal wall in GIST patients prone to necrosis and ulceration; Certain positions may make fluid and air enter the necrotic area from the intestine (C). When patients changeposition (D), based on the Bernoulli principle, atmospheric pressure (P2) becomes equal to the pressure of gas and fluid in the necrotic area (P1 + P3).Consequently, fluid and gas from the necrotic area is unable to flow into the bowel lumen.The atmospheric pressure (P2) and fluid pressure (P3) in the bowel lumen are equal to the gas pressure of the necrotic area (P1), so the fluid from the bowel lumen is unable to flow out from the necrotic area (E).
(TIF)

S5 Fig Representative CT images and histological examinations of GIST with bubbles in the rectum. (A-C) Enhancement of tumor in the rectum with multiple bubbles within the mass (white arrow); (D) The fistula (white arrow), fistula opening (white arrowhead), and necrotic cavity (black arrowhead) in the dissected specimen; (E) Histopathological examinations using H&E staining showed the spindle cells (magnification, x200); (F) Immunohistochemical staining showed positive for CD117 (magnification, x200).
(TIF)

S6 Fig Representative CT images and histological examinations of GIST with an air-fluid level detected in gastrointestinal barium examination. (A, B) Air-fluid levels were observed in the gastrointestinal barium examination (black arrow) (C). CT contrast-enhanced scan showed enhancement of the mass and a central air-fluid level and (D) oral barium(white arrow) without being able to enter the mass (E). The mass specimen presented the ulcer of mass (white arrowhead) and fistula opening from the inner wall of the small intestine. Histopathological results by H&E staining showed the spindle cells (thin white arrow) and epithelial cells (thin black arrow) (C, magnification,x100) (D, magnification, x200).
(TIF)

S7 Fig Representative CT images and histological examinations of GIST with the smaller air-fluid level in the descending duodenum. (A) Small gas-fluid level on CT images (white arrow); (B) CT contrast-enhanced scan showed heterogeneous enhancement of the mass; (C) GIST in the descending duodenum (white arrowhead) and the mass invaded the common bile duct (white arrow), and the upper sections were expanded (D). The Portal vein (white arrow) was invaded and compressed by the mass; (E). Histopathological results by H&E staining showed the epithelial cells (magnification, x200) and (F) spindle cells (magnification, x200).
(TIF)

S1 Data Control group. (XLSX)

S2 Data Experimental group. (XLSX)

## Abbreviations

CT: computed tomography
GI: gastrointestinal
GIST: gastrointestinal stromal tumor
HPFs: high-power fields
IHC: immunohistochemistry
IM: Imatinib mesylate
MD: maximal diameter
MIP: maximum intensity projection
MRI: magnetic resonance imaging
MRP: multi-planar reconstruction
NIH: National Institutes of Health
PET: position emission transverse tomography
PSM: propensity score matching
T-B sign: Torricelli-Bernoulli sign
