# Supplementary figures and images for: Clinicopathological characteristics and prognosis of gastrointestinal stromal tumors containing air-fluid levels
Source: PLoS One. 2021 Dec 17;16(12):e0261566. doi: 10.1371/journal.pone.0261566 (PMC8682903; doi:10.1371/journal.pone.0261566)

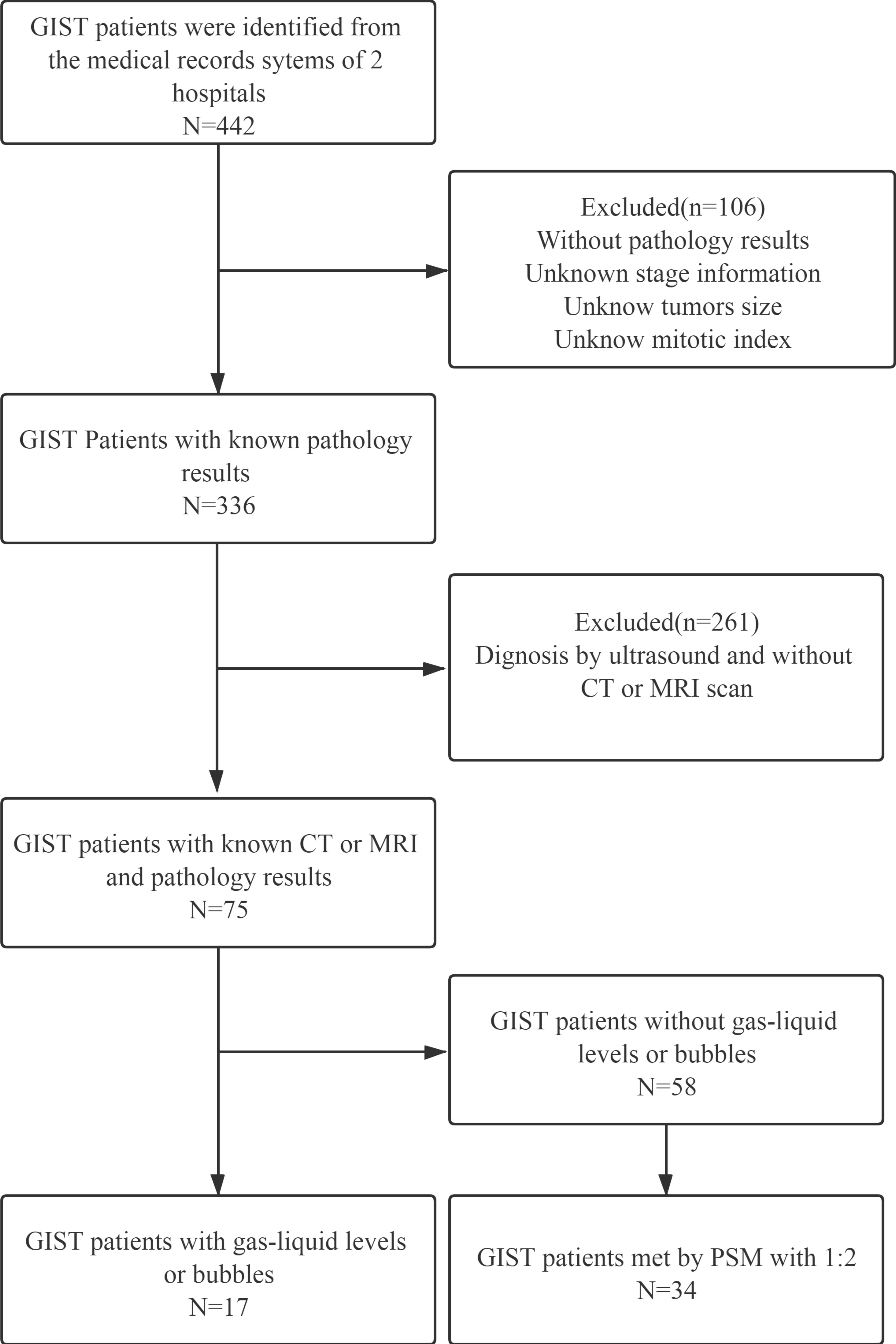

Supplement: S1 Fig — (PDF) [file pone.0261566.s001.pdf]

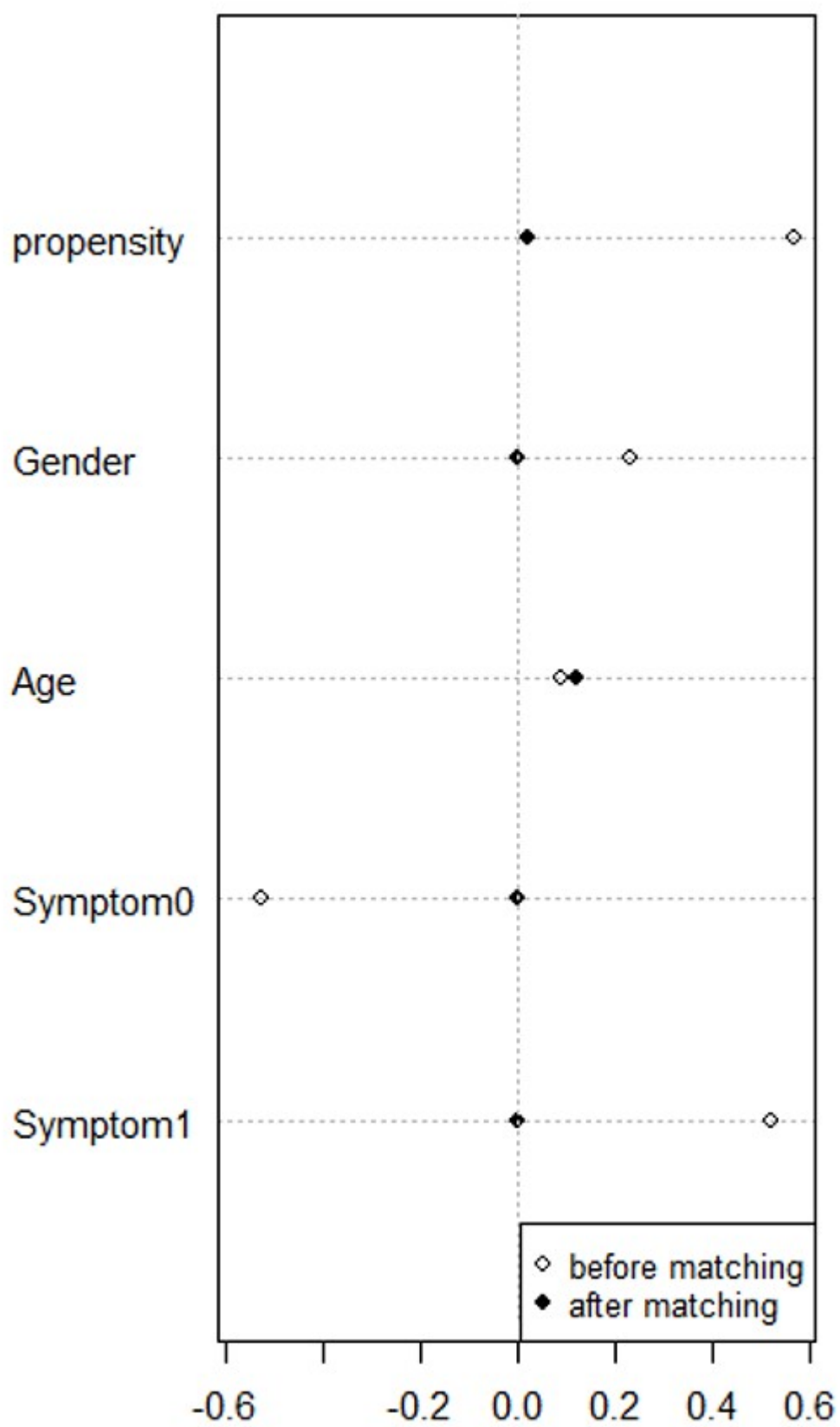

Supplement: S2 Fig — (PDF) [file pone.0261566.s002.pdf]

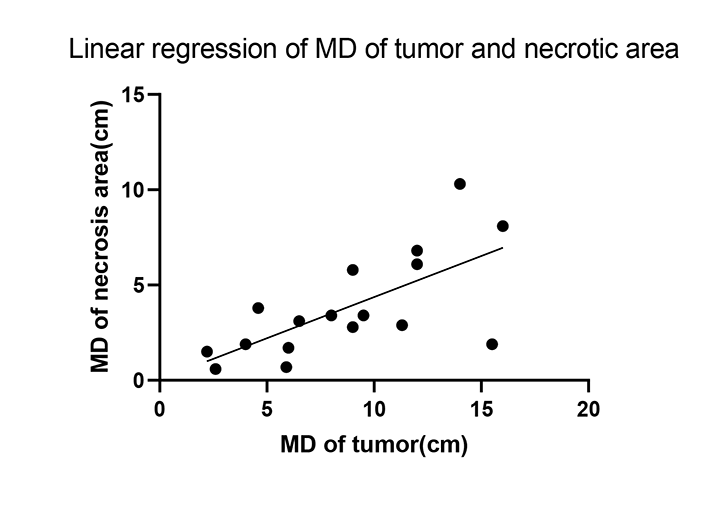

Supplement: S3 Fig — The analysis showed a significant correlation between tumor size in maximum diameter and necrotic area in GIST patients with air-fluid levels. (TIF) [file pone.0261566.s003.tif]

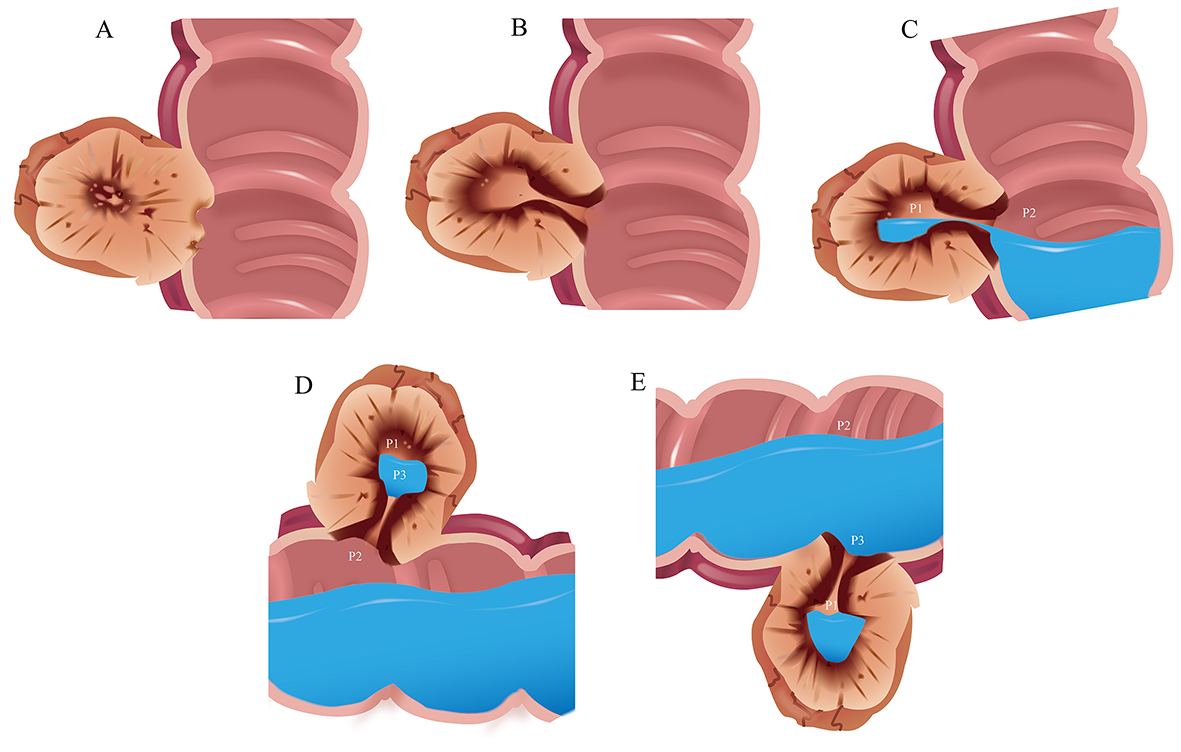

Supplement: S4 Fig — (A, B) Fistula formation between the tumor and the intestinal wall in GIST patients prone to necrosis and ulceration; Certain positions may make fluid and air enter the necrotic area from the intestine (C). When patients changeposition (D), based on the Bernoulli principle, atmospheric pressure (P2) becomes equal to the pressure of gas and fluid in the necrotic area (P1 + P3).Consequently, fluid and gas from the necrotic area is unable to flow into the bowel lumen.The atmospheric pressure (P2) and fluid pressure (P3) in the bowel lumen are equal to the gas pressure of the necrotic area (P1), so the fluid from the bowel lumen is unable to flow out from the necrotic area (E). (TIF) [file pone.0261566.s004.tif]

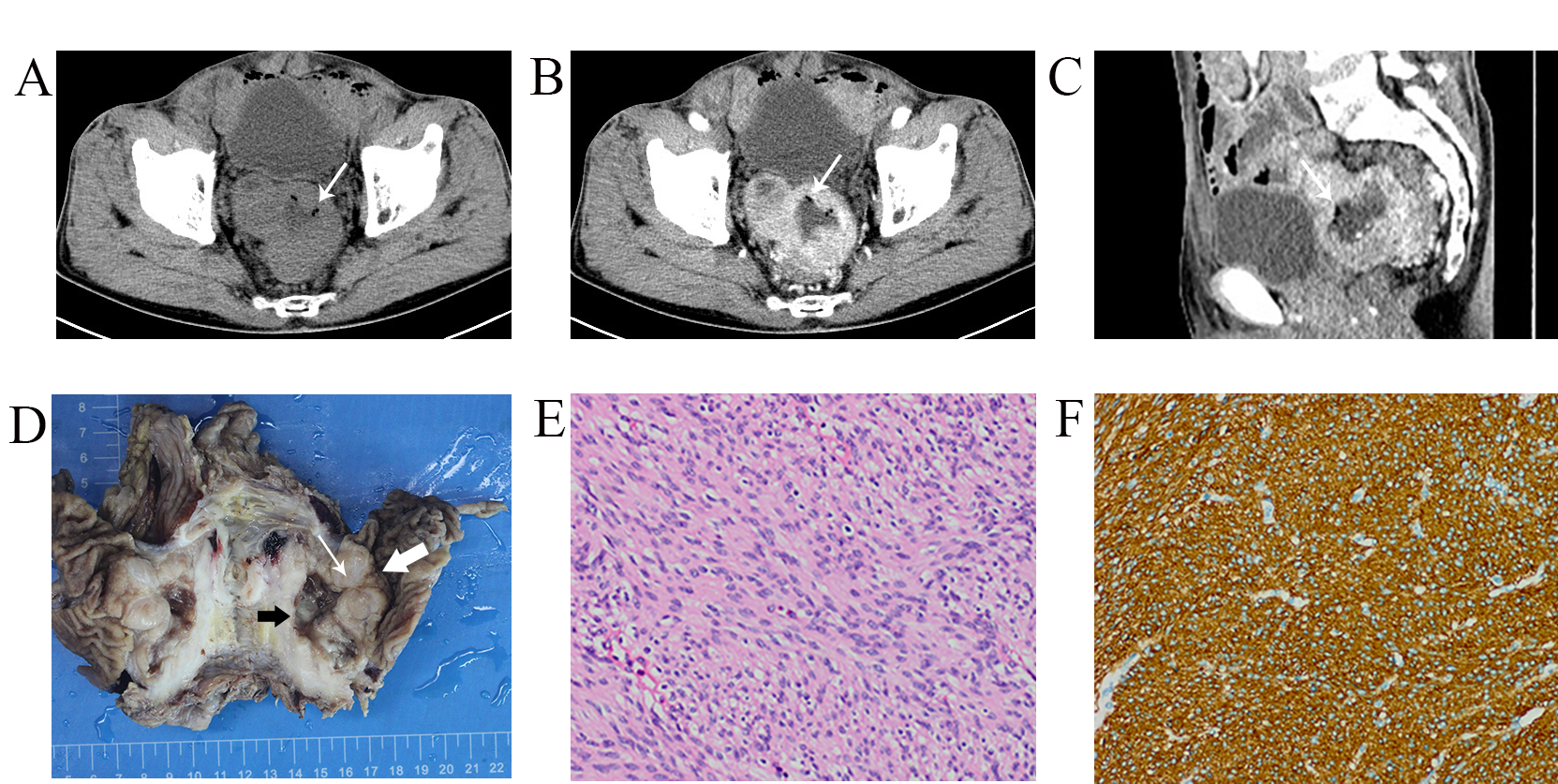

Supplement: S5 Fig — (A-C) Enhancement of tumor in the rectum with multiple bubbles within the mass (white arrow); (D) The fistula (white arrow), fistula opening (white arrowhead), and necrotic cavity (black arrowhead) in the dissected specimen; (E) Histopathological examinations using H&E staining showed the spindle cells (magnification, x200); (F) Immunohistochemical staining showed positive for CD117 (magnification, x200). (TIF) [file pone.0261566.s005.tif]

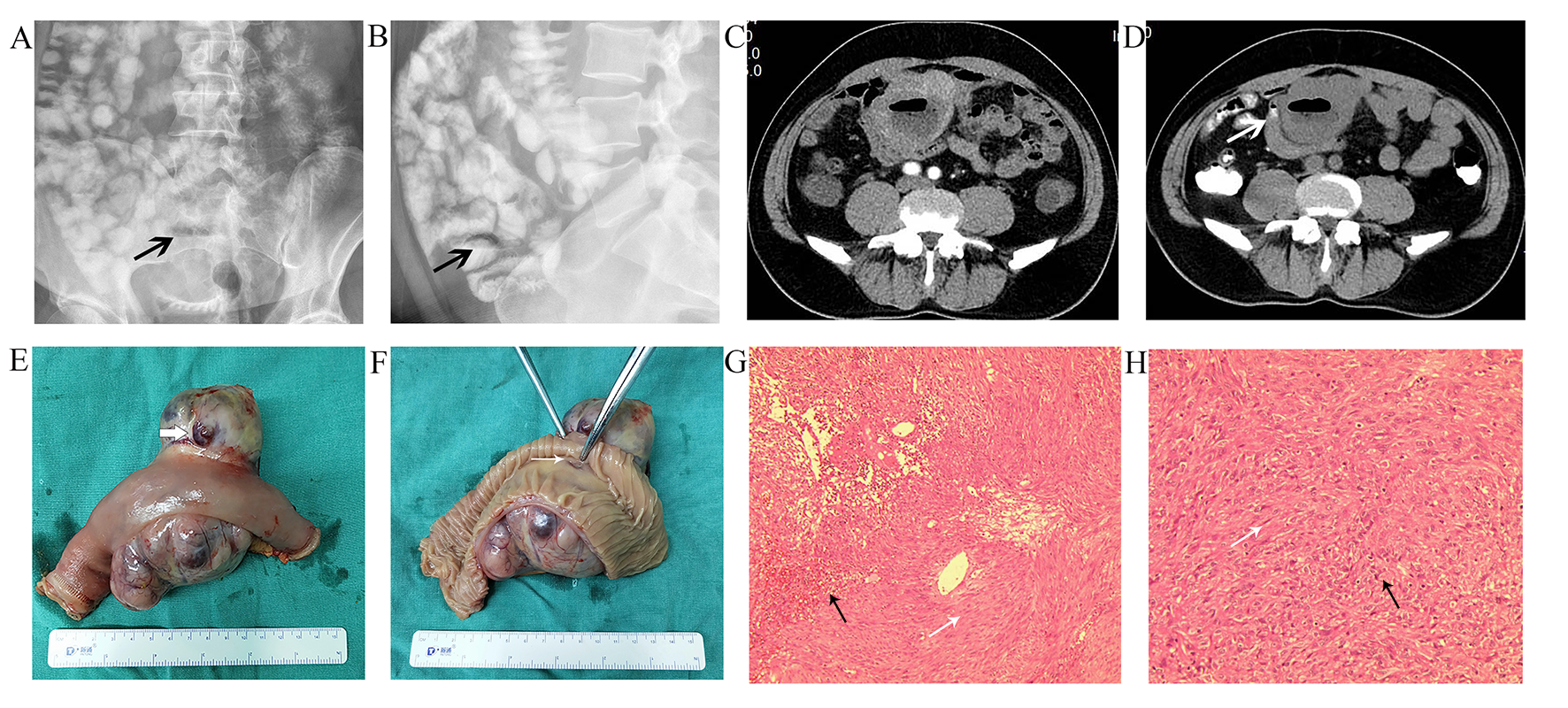

Supplement: S6 Fig — (A, B) Air-fluid levels were observed in the gastrointestinal barium examination (black arrow) (C). CT contrast-enhanced scan showed enhancement of the mass and a central air-fluid level and (D) oral barium(white arrow) without being able to enter the mass (E). The mass specimen presented the ulcer of mass (white arrowhead) and fistula opening from the inner wall of the small intestine. Histopathological results by H&E staining showed the spindle cells (thin white arrow) and epithelial cells (thin black arrow) (C, magnification,x100) (D, magnification, x200). (TIF) [file pone.0261566.s006.tif]

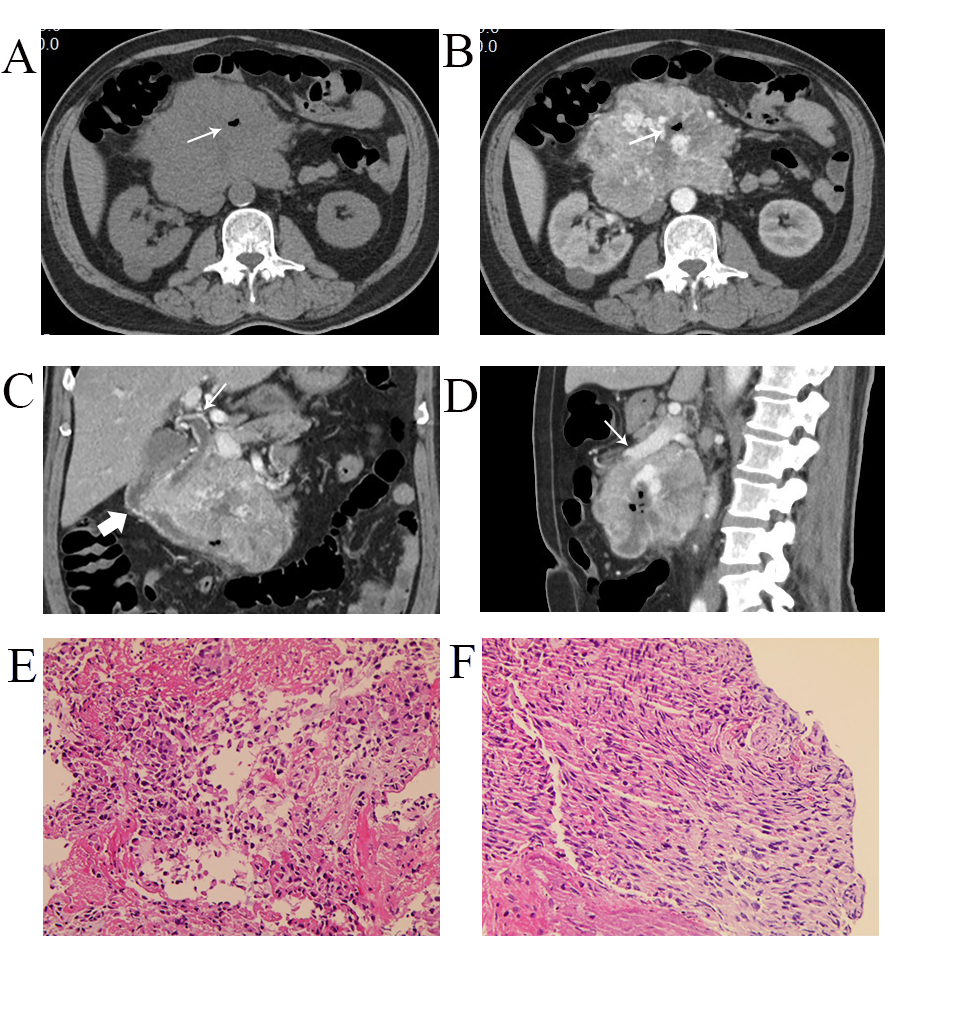

Supplement: S7 Fig — (A) Small gas-fluid level on CT images (white arrow); (B) CT contrast-enhanced scan showed heterogeneous enhancement of the mass; (C) GIST in the descending duodenum (white arrowhead) and the mass invaded the common bile duct (white arrow), and the upper sections were expanded (D). The Portal vein (white arrow) was invaded and compressed by the mass; (E). Histopathological results by H&E staining showed the epithelial cells (magnification, x200) and (F) spindle cells (magnification, x200). (TIF) [file pone.0261566.s007.tif]
